# Information Needs of Women Affected by Endometriosis and Their Environment: Qualitative Results from Participatory Workshops

**DOI:** 10.3390/healthcare14040449

**Published:** 2026-02-11

**Authors:** Nina Lorenzoni, Elisabeth Nöhammer

**Affiliations:** Department of Public Health, Health Services Research and HTA, UMIT TIROL—Private University for Health Sciences and Health Technology, 6060 Hall in Tirol, Austria; elisabeth.noehammer@umit-tirol.at

**Keywords:** endometriosis, information needs, chronic diseases, women’s health, participatory workshops

## Abstract

**Highlights:**

**What are the main findings?**

- Patients, social networks, and healthcare providers require comprehensive, life-stage-specific information on endometriosis, including disease progression, treatment and psychosocial support options, plus legal/financial guidance.
- Participatory workshops identified critical gaps in currently available information material, emphasizing the need for clear, empathetic, and evidence-based content tailored to diverse audiences (patients, their social networks and healthcare providers).

**What are the implications of the main findings?**

- A centralized, co-created information ecosystem with modular, updatable digital platforms could address information gaps, improving endometriosis literacy, trust, and care coordination.
- Integrating patient expertise into resource development enhances relevance and trust, providing a transferable framework for other chronic conditions.

**Abstract:**

**Background/Objectives**: Endometriosis affects ~10% of women, causing chronic pain, reduced quality of life, and, often, infertility. As endometriosis literacy and awareness are low in society and among health care providers (HCPs), patients are often on their own. The aim of this study was to identify information needs of patients, their social networks and HCPs based on the perspective of the patients as experts. **Methods**: Four participatory workshops (two online, two in-person) with 45 Austrian women (ages 20–50) diagnosed with endometriosis were conducted. Using pinboards for target groups (patients, partners/social networks, HCPs) and reviews of existing materials, we explored gaps and co-created ideas for information material and dissemination. **Results**: Participants emphasized the need for comprehensive, up-to-date, evidence-based information on disease progression, multimodal treatment options, and life-stage-specific topics such as fertility, surgical aftercare, or menopause. The necessity of legal and financial guidance (e.g., disability rights or prescription fee exemptions) was highlighted, alongside clear pathways to specialized care. Some of the existing materials or contents were criticized as outdated, inconsistent, or difficult to read. For their social networks, participants requested simple, empathetic materials explaining disease chronicity, intimacy/fertility challenges, and practical support during pain episodes. HCPs were urged to update clinical knowledge, proactively address symptoms, validate pain experiences, and be transparent about treatment side effects and psychosocial burdens. **Conclusions**: A centralized, co-created, and easily accessible information ecosystem could support patient autonomy, improve trust, and reduce diagnostic delays. Strengthening self-help groups and life-stage-tailored information are critical to improving self-management and mitigating endometriosis’s socioeconomic impact.

## 1. Introduction

Endometriosis is a chronic and inflammatory disease that is characterized by endometrial-like tissue growing in other parts of the body, outside of the uterus [1]. It affects around 10% (190 million) of women and girls of childbearing age worldwide [2], with the number potentially higher due to underreporting or remaining undiagnosed [3]. Endometriosis is associated with chronic pelvic pain, severe pain during periods, pain-related problems during sexual intercourse, painful bowel movements and/or urination, bloating, nausea, fatigue, and, sometimes, depression, anxiety, as well as infertility [4]. Due to their severity and variety, the symptoms can have a negative impact on all areas of life (social life, relationships, work, school) [5].

Women living with endometriosis thus often face a significantly reduced quality of life, higher rates of depression, and strained intimate relationships. Their medical condition can also limit their ability to engage in daily and social activities, leading to loss of productivity and consequently income. Furthermore, endometriosis imposes substantial direct and indirect healthcare costs [6,7,8]. Research also highlights a stronger association between endometriosis and complications during pregnancy and childbirth [9,10,11].

Despite its prevalence, there is still a marked diagnostic delay. Depending on the geographical region [12,13], the timespan between symptoms and diagnosis varies between less than a year and 12 years [1,7,12,13,14,15,16]. This is due to the large variety of symptoms [16] and stigmatization of menstrual health, but also to partly limited information about the disease on a societal level [17], among medical personnel, and those affected.

Some women are unaware that menstrual pain is treatable [18] and that excessive pain is not normal. On average, patients seek treatment 2.3 years after the first symptoms [14], and need around seven visits to their primary healthcare provider (HCP) before being referred to specialists [12]. In addition, the brief time slots for HCP visits might hinder adequate assessment [19], and even the regular checks at gynecologists often do not systematically address endometriosis symptoms [20]. Specialized endometriosis centers exist in some countries but are unevenly distributed and difficult to access without a referral. As a result, early detection and consistent treatment remain challenging despite formal access to care [18]. This may be the reason why 3/4 report at least one misdiagnosis [14], and studies highlight a lack of information given by HCPs on the impact of endometriosis on intimate relationships [21], choice of therapy, dealing with pain, and the desire to have children [22].

Information thus plays a crucial role in decision-making and doctor–patient communication, but also in coping with the disease and engaging in self-management. Many affected individuals even report that they obtain information from influencers because they feel that this is the only source of information that is realistic and relevant to their lives [23]. Thus, low-threshold, evidence-based information on endometriosis is needed to empower those affected as patients, their social network, and caregivers. To best target and cover these information needs, the participation of patients is recommended [5]. This is especially relevant as the current knowledge on information needs of women with endometriosis mainly refers to medical topics [24] or only to information needs of patients, excluding their social networks, or HCPs.

Therefore, this paper aims to cover information needs holistically. Via participatory research, it investigates the patients’ suggestions regarding information for themselves, their social networks, and their medical caregivers. Involving women with endometriosis in the research process allows for uncovering unmet (information) needs and creates a space where they can share ideas for improved support. Involving them in the research process recognizes their expertise and experience. This can help to reduce stigma, raise awareness of the condition and improve the quality of care and support for women with endometriosis [24,25].

## 2. Materials and Methods

The study was conducted in Austria, where the average time from suspicion to diagnosis is currently 6.6 years [26]. Specialized endometriosis care is beginning to become available, but only in larger cities and without any established quality control measures in place. Self-help groups are highly active, and there has been a public media campaign on endometriosis to inform society regarding the existence of the disease, making it the ideal region for discussing information needs.

As described above, we opted for a participatory research approach. Participatory research serves as an overarching concept encompassing methods, frameworks, and principles that actively involve individuals with lived experience in the research process. This shifts the focus from researching about communities to researching alongside them, ensuring their voices and perspectives shape the process [27]. Participatory research approaches like co-creation with patients can also promote acceptance of health promotion and health information [28].

Therefore, we chose co-creative workshops as a method to best elicit and integrate the perspectives and life realities of the participants. Our approach thus aligns with the interpretive paradigm (subjective, but status-quo-oriented), focusing on actors’ subjective constructions of their current realities, while its participative design also allows for radical humanist elements (subjective, but focusing on societal and structural change) by reflecting on empowerment and power dynamics [29].

### 2.1. Participant Recruitment and Study Preparation

The study was approved by the Research Committee for Scientific and Ethical Questions (RCSEQ) of UMIT TIROL—Private University for Health Sciences and Health Technology (Nr. 3288). We conducted four 4 h workshops (two online, two in person—one in the western and one in the eastern part of Austria) with 10–15 participants each. By providing the workshops as online and on-site sessions in different parts of the country and offering free childcare during the workshops in presence, participation was low-threshold. We selected the workshop locations based on accessibility and suitability, opting for hotels or public spaces with appropriate meeting facilities that were rented specifically for the event. Moreover, participants received EUR 100 as compensation for their participation based on the recommendation of the funder [30].

Participants were recruited via social media channels of the funder, a health-focused university (affiliation of the authors), personal contacts, the project website, announcements at gynecologists and specialists for endometriosis, a public conference on open innovation in science, self-help groups and an endometriosis association (Verein Endometriose Österreich). They had to be available for one of the workshops and prepare for discussion in advance by screening four brochures on endometriosis (details below). They received these four brochures via email, together with details regarding the workshop location, informed consent, and payment information.

The brochures were retrieved via a Google online search, as this would also be the common way someone affected by endometriosis would do their information seeking. On the first 15 result pages, we found 20 brochures, flyers or information material on endometriosis in German for non-medical staff. We then excluded six brochures that consisted of only one or two pages. The remaining 14 brochures (overview in Table A1 in the Appendix A) were reviewed within the team to assess their suitability as discussion material for the workshops. Eight brochures were ultimately selected for use in the workshops. In each workshop, four brochures were reviewed. The same set of four brochures was discussed in two workshops, ensuring that each brochure was addressed twice in the workshop series. Length, depth, and publisher of the included materials varied, which made a fair distribution of preparation time possible.

The study participants were 45 Austrian women aged between 20 and 50 years with a self-reported diagnosis of endometriosis. The number of participants per workshop was chosen to enable in-depth, participatory discussions. This approach is supported by the concept of information power [31], given the specificity of the sample and the thematic consistency observed across all workshops. At the time of the study, the participating women had been diagnosed with endometriosis between six months and 18 years prior (Table 1).

### 2.2. Workshop Outline

The workshops were used as a co-structured space to discuss the information needs from the patients’ perspective and to discuss what partners, families, other important people, and medical staff should know about endometriosis. After (1) a round of introductions, repeating project information and checking informed consent, the participants were (2) presented with quotes from other women with endometriosis available in the literature, describing their experiences with the disease and the path to diagnosis. The participants were asked to select quotes that they felt were relevant to them and to elaborate on their own experiences based on these quotes. This approach was chosen as a start into the topic and to create a personal environment where participants felt psychologically safe.

Then, (3) four pinboards were set up around the room for the respective information target groups: “*Myself*,” “*Partner*,” “*Environment*—*Friends*, *Workplace*,” and “*Medical Staff*.” The participants were free to add their own content (i.e., information needs) to the pinboards. After the free collection of topics, these were discussed in the group and elaborated on in more detail. After a break with refreshments, we (4) discussed four existing endometriosis information brochures per workshop (eight in total; length, thematic focus, and publisher varied) and their advantages and disadvantages to develop specific ideas for their improvement and discuss (better) dissemination strategies and necessary changes in the healthcare system.

To protect the open discussion atmosphere and to reduce inhibitions, we decided not to record the workshops. The workshops were moderated by two or three members of the project team. This gave one person the opportunity to fully concentrate on moderation while the others took extensive notes, pictures of finished flipcharts and pinboards, and assisted with organizational issues. None of the researchers or moderators had a prior relationship with the participants, nor did any team member have personal experience with endometriosis, to avoid bias. After each workshop, the team conducted structured debriefings to reflect on both the content (e.g., consistency in data collection, emerging themes) and the emotional impact of engaging with participants’ experiences. These discussions allowed us to critically assess our facilitation techniques and analytical interpretations.

### 2.3. Data Analysis

To systematically analyze the data, we employed a participatory thematic analysis approach, integrating both in situ clustering with the participants in the workshops and post-workshop refinement by the project team.

During the workshops, participants contributed to four themed pinboards representing key target groups: “*Myself*,” “*Partner*,” “*Environment*—*Friends*, *Workplace*,” and “*Medical Staff*.” This open-ended format allowed participants to freely identify and prioritize informational needs without predefined constraints. After the initial collection of ideas, the group collaboratively discussed, refined, and clustered the content. The pinboards representing the target groups (patients [*myself*], social networks [*partner*, *environment*], HCPs [*medical staff*]) served as predefined overarching themes. The subcategories emerged organically from the participatory discussions within each target group. For the brochure review, we deliberately did not establish predefined criteria or guidelines. Imposing such standards would have directed participants’ feedback in a specific way, whereas our goal was to center patients as experts in identifying their own informational needs. Again, the participants discussed and clustered the content from the discussions on flipcharts and pinboards, assisted by project team members in case required. The in-workshop clustering served as an early form of member checking, as participants collectively agreed on the relevance of themes. No coding software was employed, as the clustering process was participatory and conducted during the workshops with the participants. After the workshops, NL and EN cross-referenced findings across pinboard and flipchart content, field notes, and debrief reflections to identify recurring topics, patterns, and gaps in the available information. Regular team discussions were held to resolve discrepancies and refine thematic categories. Disagreements were addressed through consensus-based dialogue.

## 3. Results

The results presented below are structured regarding the information needs perceived by the patients for themselves, their social networks, and medical personnel. They were thematically structured in the workshops in part (3), with more detail provided in part (4). Direct quotes are added in italics and are verbatim transcriptions written down in real time by the project team members during the workshops. Table 2 provides an overview of the overarching themes and identified subcategories in the workshops.

### 3.1. Information Needs of Women Suffering from Endometriosis

The participants reported extensive information needs. Some stated that they were not aware of the existence of the disease and only came across it when they were conducting research on their symptoms. Many of those affected said that they would have liked more information and education about the chronic nature of the disease, its progression, and the need for continuous treatment: “*It’s not enough to go to the doctor once or have one surgery, or take the pill [birth control pill, note by project team] and then everything is fine. It is a chronic disease and should be treated holistically and evaluated on an ongoing basis!*” Therefore, the need for information about alternative treatment methods through complementary and natural medicine was expressed*:* “*Getting informed about the variety of treatment options, including surgery and medication, as well as other ways to alleviate symptoms, and also address coping strategies.*” Some even found the diagnosis itself a relief: “*It meant I knew I wasn’t crazy and that I could be helped.*”

Information tailored to different life stages and priorities was another need expressed by the participants, especially during the reproductive phase and menopause. Specific information on fertility treatments, pregnancy and breastfeeding with endometriosis was also in demand, as this seems to be very rare at present. Adequate health information should be available on topics like treatment options and their actual chances of success, including surgery and post-surgical care, pharmacological options, nutrition, and interdisciplinary treatment methods. One participant commented: “*Information about aftercare or an integrated care perspective is completely neglected. You have surgery and then you’re discharged, and that’s it.*”

Participants furthermore expressed a demand for information about specialized doctors and facilities to be readily available, as many of those affected had to change doctors several times before receiving a diagnosis and treatment. Even officially recognized specialization centers sometimes fall significantly short of expectations in terms of expertise. Self-help groups and endometriosis associations often function as a substitute for this wished-for point of contact for exchange and support. Participants used and referred to the terms “association” and “self-help groups” interchangeably, as both serve as key sources of information and peer support. In some federal states, local self-help groups are particularly active, while others rely on the national association as primary contact. Both were valued as well-informed resources for exchange and guidance. As they are limited in terms of time and financial resources, the participants spoke out in favor of strengthening them as reliable, low-threshold points of contact.

Financial difficulties were discussed, as in some cases it is necessary to reduce working hours due to illness, and at the same time, additional treatments are not covered by health insurance. Legal information was also identified as necessary, such as the possibility of prescription fee exemptions, insurance coverage for the costs of hormonal contraceptives, or applications for disability status. Also, layoffs were described as common due to frequent sick leaves because of pain episodes, especially when patients decide not to disclose their condition at the workplace. Some endometriosis patients therefore decide to communicate their condition and opt for official disability status to ensure higher legal protection against layoffs. Several workshop participants were not even aware of this possibility, despite having been diagnosed with endometriosis years ago, and learned about it in the workshop discussions. Many of the others described how they had found out about this legal option relatively late and only through their own research. Therefore, “*In addition to medical information, there should also be a kind of contact point where people can go and get help and tips for their everyday life with endometriosis.*”

### 3.2. Information Needs of Social Networks

The participants saw it as a fundamental problem that endometriosis is still either completely unknown to most people in their environment (“*Many haven’t even heard the term yet!*”) or its far-reaching effects are not clear. Although information needs differ depending on the setting, e.g., family/partner/work, in general, basic facts about the condition itself and its chronicity should become common knowledge: “*People need to understand that this is a chronic illness that won’t get better overnight, and there is no cure for it.*” Faced with low societal knowledge, they are given the responsibility to explain everything to their network, which leaves them highly frustrated and worn out. Greater awareness of the disease would reduce the burden of explaining it, as one participant stated: “*As someone suffering from endometriosis, I am not responsible for educating others about it!*”

As a potential alleviation and to increase general societal knowledge, parents and teachers should inform adolescents about which pain intensity is (ab)normal during the menstrual cycle. Through greater awareness of endometriosis and its consequences, consideration for the requirement of more flexibility at work or school is hoped for. This is needed regarding medical appointments and understanding of reduced performance and restricted possibility to participate in social life during pain phases.

Also, all significant others should be informed about the mental burden of the disease. Open, trustful communication and empathy are desired for: “*They should understand that the pain is far worse than I can possibly express! It feels like lava in my belly!*” Another participant stated: “*Don’t belittle the complaints and feelings of those affected just because you can’t or don’t want to deal with them.*” Often, social networks want to help but do not know how. Partners need easy-to-understand information on how they can help and regarding the impact of endometriosis on reproductive health, family planning and sexual activity. Self-help groups for partners of patients were suggested.

### 3.3. Information Needs of Medical Personnel

The major information need of medical personnel, according to the workshop results, is knowledge about the disease itself. Often, participants stated having a markedly better knowledge about the disease than even gynecologists, and that this lower knowledge is resulting in a diagnostic delay and patients’ loss of trust in the healthcare system. As one participant put it: “*I lost faith in medicine and never regained it.*” Moreover, participants reported feeling very tired of having to look for information themselves. Own research is even reported as necessary to ensure being taken seriously: “*I learned the terminology so that I could prepare myself for health encounters.*”

Participants highlighted that they felt that medical personnel, except in specialized endometriosis centers, did not possess adequate knowledge of the multifaceted symptoms and subsequent patient needs or treatment options. They also highlighted that there seems to be no or not enough quality control regarding medical personnel who were presenting themselves as experts on endometriosis. The participants suggest that information about endometriosis should be included in all healthcare curricula, as the symptoms are often not only gynecological. Moreover, addressing it in all healthcare professions could improve interdisciplinary treatment. Ideally, endometriosis information packages co-created with patients are available for all HCPs.

Moreover, medical staff need to be better informed about how to communicate with patients in general, and with those affected by endometriosis in particular. Participants reported not feeling taken seriously as symptoms were downplayed or dismissed as psychological. In addition, pelvic pain was often presented as normal. “*I suspected it might be endometriosis, but if the doctor says it isn’t, then you believe it,*” described one individual. According to the participants, the fact that pelvic pain and period pain are often presented as normal also makes diagnostic discussions more difficult. Many women, especially younger women, often lack information about what is „normal”: “*Doctors should actively ask about symptoms and not just say, ‘Is everything okay?’*” Moreover, treatment plans should be carefully presented. “*When doctors say ‘Let’s try this’—statements like this make patients feel as if they are being used for experiments!*”

The participants also highlighted that medical personnel should possess the latest information on treatments and their success rates. Most especially, they wanted doctors to know that pregnancy is not necessarily a cure, as some participants got this recommendation as a treatment method. However, this is not evidence-based, and many endometriosis sufferers have fertility problems due to the disease. Some patients may not see pregnancy as a priority either. “*Don’t just focus on reproductive ability, but also on the fact that women want to live pain-free!*”

It would be desirable for HCPs to have more information on pain management. Some participants reported that HCPs they visited attributed side effects of pain treatments to (non-existent) substance abuse. This is because high doses of painkillers affect the liver, so information on the side effects of treatments is required. In addition to purely medical issues, those affected would like health care providers to be aware of the far-reaching psychosocial and socioeconomic side effects that endometriosis entails and for these to be addressed in consultations with doctors or through psychosocial support. Pressure and socioeconomic side effects of the disease, such as relationship problems, layoffs/reductions in working hours due to frequent sick days and the resulting financial issues, are often not considered in endometriosis care.

Doctors should also be aware that such a diagnosis requires a great deal of information, which they might not have the time to deliver in complete depth. They should therefore not only take time to answer questions but also know about the information material they can refer to: “*The brochures should be handed out by family doctors or gynecologists at the latest. But that’s not how it works in reality. As a patient, I have to take the initiative and look for information myself!*” Often, self-help groups and seeking information on their own seemed necessary to supplement the lacking information.

### 3.4. Information Delivery

The brochures reviewed during the workshops received mixed feedback. While some were considered outdated and visually unengaging, others were seen as helpful. A frequent point of criticism was the imagery used, which participants found partly unrelatable or unhelpful. Shorter brochures with less content were considered suitable for patients who had only recently been diagnosed, as they offer an easier introduction to the topic. However, participants mainly expressed a desire for more detailed and comprehensive information to address their broader needs. There was also a request to cite original sources and scientific references to enhance the credibility and transparency of the information provided. Participants noted that certain phrases, such as the suggestion to “prepare well for conversations with medical staff’, could inadvertently place undue responsibility on those affected. Similarly, the common advice “exercise is beneficial” was criticized for potentially implying that pain can simply be alleviated through physical activity, again shifting responsibility onto individuals. Additionally, key topics, including nutrition, disability assessment and prescription charge exemptions, were either absent or inadequately addressed. A significant concern was the lack of accessibility: none of the materials were designed to be easily readable by people with visual impairments. Participants clearly expressed the need for simpler, clearer language, multilingual options and inclusive formats that cater to diverse audiences, including potential caregivers of people with special needs. Overall, there was a desire for practical, everyday-oriented and supportive materials that empower those affected and their networks.

## 4. Discussion

The aim of the study was to evaluate the information needs of endometriosis patients, their social contexts and medical personnel based on the perspective of the patients as experts. This was done in four participatory workshops by evaluating existing information and having participants co-create lists of information needs together with suggestions for improvements in existing information material. This approach provides a more comprehensive understanding of systemic challenges and opportunities for support. It served as a combination of participation, empowerment and network-fostering opportunity.

Information needs arise from being affected by the disease, the lack of knowledge in society and thus direct social relationships, plus due to medical curricula not fully covering reproductive and female health topics.

### 4.1. Information Needs of Women Suffering from Endometriosis

Patients diagnosed with a chronic illness often experience their illness as a stressor and develop mechanisms for coping with it [32,33]. A common strategy when faced with issues that come with a chronic condition, such as endometriosis, is information seeking. While at the beginning the main question centers on the causes of the symptoms, after diagnosis, there is uncertainty about what to do next [34]. The perceived helplessness when trying to manage unexplained pain is high [35], which is why many sufferers feel the need to educate themselves. This self-education is central to managing symptoms and building resilience [32], but also as preparation for health encounters [18]. Sources of information used for self-education include written material, like books, brochures and online research, but also exchange with other affected persons via endometriosis associations, self-help groups and social media [18,32].

Just as reported by Senyel et al. [24] summarizing four studies, women with endometriosis want information on

- the disease itself (incl. causes, progression and the complete consequences for all areas and phases of life, i.e., relationships, pregnancy/infertility/menopause, pain and disease management);
- its therapy options (incl. physical and psychological, especially surgery);
- support available in healthcare;
- self-help strategies;
- how to communicate with their social networks;

Ideally, this information is provided by HCPs. Roomaney and Kagee [32] found that real-life information, i.e., other patients’ experiences, was mentioned as relevant, especially for those who knew no other affected person. In our study, this was discussed in the context of information seeking (necessity) and how self-help groups were providing the assistance the participants felt was not fully covered in the healthcare system. Participants additionally highlighted the relevance of legal information, an aspect not focused on in the literature so far. They also made it very clear that receiving high-quality information, communicated in a compassionate way, was not always the case.

Based on the work done on various medical conditions, Senye et al. [24] suggest that information needs vary based on disease progression, life phases, needs, culture, and personal living conditions and choices. This was confirmed by the participants in our workshops, except for culture. As a new topic, however, the respondents highlighted that information material on endometriosis should also be available for individuals with potential physical or cognitive limitations, as well as for their caregivers, and be available in Braille, audio format, and easy language.

Not only accessibility but also the quality of information about endometriosis poses challenges. As the workshops showed, some of the information provided in the brochures was already out of date. This is why the internet would be a useful medium for providing information, as websites are easy to update. Social media can also help to publish new information rapidly and increase awareness, exchange and self-help (often covering, e.g., nutrition, herbs, and exercise/yoga), but also carries a high risk of misinformation [18]. However, quality assurance is more difficult to implement online. Hirsch et al. [36] analyzed 54 websites on endometriosis in terms of quality and readability. None of the analyzed sites were easy to read; 83% were at college level or higher, often incomplete, inaccurate or poorly written. Inadequate health information bears risks of physical, emotional or financial harm [36]. Contradicting information from internet research with the information received from HCPs can lead to mistrust in medical recommendations [37,38]. Independent information services such as informedhealth.org, provided by the Institute for Quality and Efficiency in Health Care, are attempting to raise awareness. However, it remains unclear which channels effectively reach those affected and what effects this has on the use of healthcare services [18].

### 4.2. Information Needs of Social Networks

Women with endometriosis perceive an empathetic and understanding environment as an important support mechanism in dealing with the disease. The frequent lack of awareness of the far-reaching effects of the disease often leads to problems in social relationships [6,39]. Most importantly, partners need to be educated regarding the effects of endometriosis on reproductive health, consequences for romantic relationships, family planning, and how to help during pain episodes. The latter is also relevant in friendships, as all those in the social networks of patients need to know that, e.g., cancelling appointments in these phases is to be expected. Therefore, several studies [6,39,40] have emphasized the need for educational materials for partners, family, friends and employers, as was underlined in the workshops. At the time of our study, no specific brochures aimed at relatives, friends, employers or partners could be found in German. In some brochures for patients, the topic “partnerships” was mentioned only briefly. However, the Endometriosis Association Germany now has a dedicated section on its website aimed specifically at relatives [41].

### 4.3. Information Needs of Medical Personnel

Medical training does not yet extensively cover reproductive health and endometriosis [42,43,44]. Some doctors report hearing about endometriosis for the first time in continuing education courses or from their patients [44]. This may be one of the reasons why most workshop participants described the path to diagnosis as challenging and frustrating, requiring multiple visits to different doctors. The arduous path to diagnosis described by the participants is consistent with the literature. In most cases, multiple contacts with HCPs [45] and referrals to specialized facilities are necessary [46,47]. According to the workshop participants, this is due to a lack of endometriosis-related education and training among many health care providers. According to them, many general practitioners are not sufficiently informed about the disease and its main symptoms [48,49] and underestimate its prevalence rate [42,43]. Training on endometriosis and its wide-ranging, diverse symptoms should therefore be included in all medical curricula and continuing medical education programs [13,50]. This ensures that the “*often cyclic and sometimes aspecific symptoms presented*” [13] (p. 528) can be correctly identified at an early stage. Initial contact with HCPs plays a key role in timely diagnosis. Another central problem is the often inadequate information provided at the time of diagnosis [6], as there is no validated, routinely used questionnaire for systematically assessing the experience of menstrual pain [43].

Participants in the workshops described searching for information on complementary medicine approaches and holistic, interdisciplinary treatment methods to manage their disease. It is precisely this kind of multimodal, interdisciplinary and patient-centered care approach that is also recommended in the literature [19,51,52]. This also means that HCPs’ expertise regarding recommendations for, referral to, or advice against complementary methods would be highly appreciated by the patients. In addition to providing comprehensive information, HCPs may profit from training to identify problems from the patient’s perspective and jointly set goals and strategies for current needs [6,53]. This would allow covering medical, psychological and quality of life aspects, and for a collaboration of various specialist disciplines (such as gynecology, pain therapy, nutritional counselling, physiotherapy and psychotherapy) to ensure holistic and individual care [51,52]. As a result, this could help reduce emergency presentations, hospital admissions and painkiller use [54], but would require resources for implementation, e.g., finances for holistic treatments [50]. Currently, the latter usually takes place in specialized centers, creating a bottleneck with long waiting times. Regional networking and the expansion of interdisciplinary, integrated care outside of specialized centers would improve endometriosis care [18]. Nevertheless, these, as well as claims regarding specialization in endometriosis care, also need quality checks and controls.

A valuable addition to the above would be communication training to acknowledge the psychosocial aspects of the disease [52]. Doctors are often inadequately trained in psychosocial aspects [50,55], do not see these as their responsibility [48], or do not have the necessary time and resources to cover them, in addition to many other demands. Time is also required to make all treatment options transparent, discuss them and show empathy for the physical and psychological stress caused by the disease [56]. Even if this is often difficult to implement in everyday life and would require a system change, it leads to high patient satisfaction [56] and, accordingly, trust in medical recommendations [57]. For the latter and to improve quality of care, research also recommends that HCPs learn to understand their (treatment plan’s) impact on patients and the skills to “*acknowledge and incorporate women’s knowledge of their bodies into medical encounters*” [58] (p. 22). The validation of emotions and pain would help to cushion the negative effects on well-being [59,60]. A lack of empathy and emotional support from HCPs can result in patients developing a long-term distrust towards the healthcare system [61]. Numerous workshop participants reported a loss of trust in the healthcare system due to the long diagnosis period and lack of treatment options and feeling not taken seriously during their medical appointments.

### 4.4. Limitations

The study relied on self-selection, as participants responded to open recruitment calls via various channels. To protect participant privacy and adhere to ethical guidelines, we did not verify participants’ self-reported endometriosis diagnoses. However, recruitment via self-help groups, the requirement of participants’ substantial commitment (i.e., time for preparing for the workshops and some participants even travelling to the workshops from different provinces), plus the depth and context-specific nature of discussions (e.g., treatments, symptom management, and diagnostic journeys) strongly indicate that all participants had lived experience with the condition. To prioritize participant comfort, we have decided not to record the workshops. While the absence of audio recordings may limit the granularity of our data, we minimized potential loss of information through real-time documentation by one or two moderators per workshop, as well as through the recurrent nature of themes across sessions. The participatory clustering of flipchart-based discussions ensured that key insights were captured and validated collaboratively on-site. Although a wide range of channels was used to recruit participants, they were rather young (mostly between 20 and 30 years old) and well educated (most had at least A-levels, many with a university degree). This may have influenced the evaluation of the brochures presented and may have led to the high level of detail in the input provided by the participants. It is also possible that the necessity to register for the study and to prepare for the workshop by reading four brochures was a deterrent for people with limited time resources or lower digital affinity. Moreover, many participants were recruited through endometriosis support groups, which may have led to an overrepresentation of participants who are more engaged or who suffer from more symptoms than other women with endometriosis. Nevertheless, these individuals would also be expected to provide more detailed feedback regarding information needs. The workshop participants had extensive knowledge and a high level of health literacy, which they acquired through self-education and exchanges in self-help groups.

## 5. Conclusions

Endometriosis patients are in urgent need of empowerment through high-quality information [62], health literacy interventions to help them understand the information they receive [23], and health promotion.

Practical Implications: All aspects of health promotion and prevention are required, including advocacy at the policy level and raising awareness in medical and public education. Involving endometriosis patients has revealed the far-reaching consequences of endometriosis in all areas of life, demonstrating the need for a comprehensive view of the disease that goes beyond the medical diagnosis. Socioeconomic side effects of the disease, such as relationship problems, reduced social participation, dismissal or reduced working hours due to frequent sick days and the resulting financial problems, are often not considered in information on endometriosis. Many participants described a loss of trust in the healthcare system, which could also be problematic in relation to other diseases. The duration of the diagnostic process, treatment options and symptoms of endometriosis are often perceived as traumatic. Participants reported losing trust in themselves and how only persistence and self-help groups helped them overcome (some) of the obstacles faced. They highlight that societal awareness needs to be raised to shorten the diagnostic delay, e.g., by already informing about endometriosis in school and in all medical curricula.

Implications for having complete and consistent information: The wide range of topics covered in the co-creative workshops reflects the need for a comprehensive and holistic approach to providing information on endometriosis. Reliable, evidence-based information resources that are easy for laypeople to understand are a necessary supplement to the information provided in doctor–patient consultations [63] and a step towards empowering those affected to manage and cope with their chronic condition [32,60]. Having complete and consistent information reduces anxiety, and being given the opportunity to ask questions, conducting independent research and discussing interventions can support patients in the management of their disease [52,62,64]. In addition to a comprehensive consultation, reliable information that is understandable to laypeople is necessary for patients and their social network to manage a chronic disease such as endometriosis. Doctors and HCPs can play an active guiding role here by referring patients to such relevant further information [24], which ideally is available in multiple languages and covers physical, social, emotional, relational, and financial questions.

Provision of Information: Although our study participants were primarily highly engaged, well-educated younger women, their ongoing difficulties accessing information suggest that systemic barriers exist even for individuals with high health literacy and advocacy experience. This raises critical concerns about the challenges faced by populations who are less engaged or less educated, who may experience even greater difficulties in navigating the healthcare system and obtaining reliable information. This further highlights the urgent need for inclusive, accessible information materials. A central information point would make it easier for those affected to obtain the information they need without having to conduct time-consuming research themselves.

Since printed material can get outdated, the (additional) creation of a digital platform might enable modular, updatable content to be tailored according to disease phase, age, topic (e.g., legal, psychosocial, medical), or audience (including a dedicated HCP section). This would allow for a flexible, adaptive information ecosystem that can evolve with users’ needs and be used for print versions. The information should be evidence-based, up-to-date and tailored to the specific needs and life stages of the target groups. Creating such high-quality information materials that are suitable for those affected, a co-creation process with HCPs, women with lived experiences and women’s health organizations is recommended [24]. Including patients in the creation of information material, also for medical personnel, is an innovative and promising approach for complex chronic diseases like endometriosis.

## Figures and Tables

**Table 1:** Participant characteristics.

Number of Participants	Registered: 50 Attended: 45
Age distribution	20–50 years
Time since diagnosis	6 months–18 years
On-site Workshop 1:	Location: Innsbruck, Tyrol (western part of Austria); many participants recruited via self-help group in Tyrol. Number of participants: 7
On-site Workshop 2:	Location: Vienna (eastern part of Austria); participants came from Vienna, Lower Austria, Styria and Burgenland; many of them were recruited via channels of Endometriosis Association. Number of participants: 11
Online Workshop 1:	Participants from different regions in Austria. Number of participants: 15
Online Workshop 2:	Many participants recruited via self-help group in Styria (south-eastern region). Number of participants: 12

**Table 2:** Key themes and subcategories identified in the workshops.

Key Themes	Topics Identified by Workshop Participants
Information needs of women suffering from endometriosis	Chronic nature of disease and need for continuous treatment Alternative treatment options through complementary and natural medicine Different information needs depending on life stages and priorities Specialized doctors and facilities Financial and legal information
Information needs of social network	Basic knowledge and awareness of disease Reducing the burden of explanation Empathy, understanding and flexibility Support groups for partners
Information needs of medical personnel	Disease knowledge and diagnosis Communication and patient interaction Treatment and pain management Psychosocial and socioeconomic impact Patient resources and support
Information delivery	Content and design Depth and credibility Language and accessibility

## Data Availability

The data that support the findings of this study are available from the first author (N.L.) upon reasonable request. The data are not publicly available due to privacy and ethical restrictions.

## Abbreviation

The following abbreviation is used in this manuscript:

HCPs	Healthcare Providers

## Appendix A. Brochures Used in the Workshops

Table: Overview of brochures used in the workshops.

Note: The brochures were used as supporting materials during the participatory workshops to explore informational gaps and co-create ideas for improved resources. The feedback provided by participants reflects their individual experiences and perspectives and is intended to contribute to the ongoing improvement of endometriosis-related information. We acknowledge the valuable efforts of all organizations and authors involved in creating these resources.

**Table A1:** Overview of the brochures used in the workshops.

Title	Publisher
a. Endometriose Basiswissen (Endometriosis Basic Knowledge)	Endometriose Vereinigung Deutschland (Endometriosis Association Germany)
b. Endometriose—Schmerzen verstehen (Endometriosis—understanding pain)	Deutsche Schmerzgesellschaft (German Society for the Study of Pain)
c. Endometriose—eine wachsende, oft verkannte Frauenkrankheit (Endometriosis—a growing, often misunderstood women’s disease)	Fachgruppe Endometriose der Arbeitsgemeinschaft für Gynäkologische Endoskopie (AGE) (Endometriosis Specialist Group of the Working Group for Gynecological Endoscopy (AGE))
d. Ernährungsinformationen Endometriose (Endometriosis Nutritional Information)	EVA—Endometriose Vereinigung Austria (EVA—Endometriosis Association Austria)
e. Informationen über Endometriose (Information about Endometriosis)	EVA—Endometriose Vereinigung Austria (EVA—Endometriosis Association Austria)
f. Endometriose—Wie führe ich ein gutes Gespräch mit meinem Arzt/meiner Ärztin? (Endometriosis—How can I have a productive conversation with my doctor?)	Endometriose Vereinigung Deutschland (Endometriosis Association Germany)
g. Leben mit Endometriose—Geprüfte Informationen und hilfreiche Tipps für Betroffene (Living with endometriosis—Verified information and helpful tips for those affected)	Gesundheitsfonds Steiermark (Styria Health Fund)
h. Mit Endometriose leben—Physische, psychische und soziale Auswirkungen (Living with endometriosis—Physical, psychological, and social effects)	Endometriose Vereinigung Deutschland (Endometriosis Association Germany)

## References

[1] Tsamantioti E.S., Mahdy H. (2025). Endometriosis. StatPearls.

[2] WHO Endometriosis. https://www.who.int/news-room/fact-sheets/detail/endometriosis.

[3] Backman I. Endometriosis. https://medicine.yale.edu/news/yale-medicine-magazine/article/endometriosis/.

[4] Olliges E., Bobinger A., Weber A., Hoffmann V., Schmitz T., Popovici R.M., Meissner K. (2021). The Physical, Psychological, and Social Day-to-Day Experience of Women Living with Endometriosis Compared to Healthy Age-Matched Controls—A Mixed-Methods Study. Front. Glob. Womens Health.

[5] Gaiswinkler S., Antony D., Delcour J., Pfabigan J., Pichler M., Wahl A. (2022). Frauengesundheitsbericht. https://jasmin.goeg.at/id/eprint/2496/.

[6] Moradi M., Parker M., Sneddon A., Lopez V., Ellwood D. (2014). Impact of Endometriosis on Women’s Lives: A Qualitative Study. BMC Women’s Health.

[7] Soliman A.M., Coyne K.S., Gries K.S., Castelli-Haley J., Snabes M.C., Surrey E.S. (2017). The Effect of Endometriosis Symptoms on Absenteeism and Presenteeism in the Workplace and at Home. J. Manag. Care Spec. Pharm..

[8] Soliman A.M., Surrey E., Bonafede M., Nelson J.K., Castelli-Haley J. (2018). Real-World Evaluation of Direct and Indirect Economic Burden Among Endometriosis Patients in the United States. Adv. Ther..

[9] Berlac J.F., Hartwell D., Skovlund C.W., Langhoff-Roos J., Lidegaard Ø. (2017). Endometriosis Increases the Risk of Obstetrical and Neonatal Complications. Acta Obstet. Gynecol. Scand..

[10] Zullo F., Spagnolo E., Saccone G., Acunzo M., Xodo S., Ceccaroni M., Berghella V. (2017). Endometriosis and Obstetrics Complications: A Systematic Review and Meta-Analysis. Fertil. Steril..

[11] Agarwal S.K., Chapron C., Giudice L.C., Laufer M.R., Leyland N., Missmer S.A., Singh S.S., Taylor H.S. (2019). Clinical Diagnosis of Endometriosis: A Call to Action. Am. J. Obstet. Gynecol..

[12] Nnoaham K.E., Hummelshoj L., Webster P., d’Hooghe T., Nardone F.d.C., Nardone C.d.C., Jenkinson C., Kennedy S.H., Zondervan K.T. (2011). Impact of Endometriosis on Quality of Life and Work Productivity: A Multicenter Study across Ten Countries. Fertil. Steril..

[13] van der Zanden M., Nap A.W. (2016). Knowledge of, and Treatment Strategies for, Endometriosis among General Practitioners. Reprod. Biomed. Online.

[14] Hudelist G., Fritzer N., Thomas A., Niehues C., Oppelt P., Haas D., Tammaa A., Salzer H. (2012). Diagnostic Delay for Endometriosis in Austria and Germany: Causes and Possible Consequences. Hum. Reprod..

[15] Staal A.H.J., van der Zanden M., Nap A.W. (2016). Diagnostic Delay of Endometriosis in the Netherlands. Gynecol. Obs. Investig..

[16] De Corte P., Klinghardt M., von Stockum S., Heinemann K. (2025). Time to Diagnose Endometriosis: Current Status, Challenges and Regional Characteristics—A Systematic Literature Review. BJOG.

[17] Hudson N. (2022). The Missed Disease? Endometriosis as an Example of ‘Undone Science’. Reprod. Biomed. Soc. Online.

[18] Neugebauer T., Schellenberg V., Allak A., Pardon S., Yilmaz-Aslan Y., Schiermeier S., Kiessling C., Schmiedl S., Dockweiler C., Brzoska P. (2025). Endometriosis– “Either Way a Tragedy”? A Qualitative Social Media Analysis of Endometriosis Perceptions in Germany. BMC Womens Health.

[19] As-Sanie S., Black R., Giudice L.C., Gray Valbrun T., Gupta J., Jones B., Laufer M.R., Milspaw A.T., Missmer S.A., Norman A. (2019). Assessing Research Gaps and Unmet Needs in Endometriosis. Am. J. Obstet. Gynecol..

[20] Brauer L., Geraedts M. (2023). Exploring Regional Healthcare Utilisation and Quality of Care for Endometriosis in Rural Areas in Hesse, Germany: A Mixed Methods Study Protocol. BMJ Open.

[21] Law C., Hudson N., Mitchell H., Culley L., Norton W. (2025). ‘You Feel like You’Re Drifting Apart’: A Qualitative Study of Impacts of Endometriosis on Sex and Intimacy amongst Heterosexual Couples. Sex. Relatsh. Ther..

[22] Remes A., Hakala M., Oikarinen A. (2023). Endometriosis Patients’ Experiences of the Counseling They Need from the Nurses through the Digital Care Pathway: A Qualitative Descriptive Study. Nord. J. Nurs. Res..

[23] Ellis K., Munro D., Wood R. (2023). Dismissal Informs the Priorities of Endometriosis Patients in New Zealand. Front. Med..

[24] Senyel D., Boyd J.H., Graham M. (2025). Informational Support for Women with Endometriosis: A Scoping Review. BMC Women’s Health.

[25] Kocas H.D., Rubin L.R., Lobel M. (2023). Stigma and Mental Health in Endometriosis. Eur. J. Obs. Gynecol. Reprod. Biol. X.

[26] Gaiswinkler S., Wahl A., Antony D., Ofner T., Delcour J., Antosik J., Pfabigan J., Pilwarsch J. (2024). Menstruationsgesundheitsbericht. https://jasmin.goeg.at/id/eprint/3814/.

[27] Vaughn L.M., Jacquez F. (2020). Participatory Research Methods—Choice Points in the Research Process. J. Particip. Res. Methods.

[28] Plunger P., Wahl A. Gesundheitsförderungsforschung in Österreich—Status Quo und Entwicklungsperspektiven. Factsheet [Health Promotion Research in Austria: The Status Quo and Development Perspectives. Factsheet]. https://jasmin.goeg.at/id/eprint/2516/.

[29] Burrell G., Morgan G. (1979). Sociological Paradigms and Organizational Analysis.

[30] Ludwig Boltzmann Gesellschaft Honorarium Guidelines for Involving Citizens and Stakeholders in Research. https://ois.lbg.ac.at/wp-content/uploads/sites/30/2025/03/OIS-Center-honorarium-guidelines.pdf.

[31] Malterud K., Siersma V., Guassora A.D. (2016). Sample Size in Qualitative Interview Studies. Qual. Health Res..

[32] Roomaney R., Kagee A. (2016). Coping Strategies Employed by Women with Endometriosis in a Public Health-Care Setting. J. Health Psychol..

[33] Conduah A.K., Essiaw M.N., Ofoe S.H. (2025). Coping with Chronic Illness: A Systematic Review of Adaptive Strategies Across Cancer, COPD, Diabetes and Heart Disease. Public Health Chall..

[34] Lunsford K., Sams A., Treise D. (2025). “We’re Just Walking Experiments”: Exploring Uncertainty Management of Endometriosis. Women’s Reprod. Health.

[35] Yoon Y., Park M.-A., Park S. (2021). Seeking Adaptation from Uncertainty: Coping Strategies of South Korean Women with Endometriosis. Res. Nurs. Health.

[36] Hirsch M., Aggarwal S., Barker C., Davis C.J., Duffy J.M.N. (2017). Googling Endometriosis: A Systematic Review of Information Available on the Internet. Am. J. Obstet. Gynecol..

[37] van den Haspel K., Reddington C., Healey M., Li R., Dior U., Cheng C. (2022). The Role of Social Media in Management of Individuals with Endometriosis: A Cross-Sectional Study. Aust. N. Z. J. Obstet. Gynaecol..

[38] Adler H., Lewis M., Ng C.H.M., Brooks C., Leonardi M., Mikocka-Walus A., Bush D., Semprini A., Wilkinson-Tomey J., Condous G. (2024). Social Media, Endometriosis, and Evidence-Based Information: An Analysis of Instagram Content. Healthcare.

[39] Grogan S., Turley E., Cole J. (2018). ‘So Many Women Suffer in Silence’: A Thematic Analysis of Women’s Written Accounts of Coping with Endometriosis. Psychol. Health.

[40] Sirohi D., Ng C.H.M., Bidargaddi N., Slater H., Parker M.A., Hull M.L., O’Hara R. (2025). Exploring Endometriosis Community Needs to Co-Create the EndoZone Digital Health Platform: A Qualitative Research Study. BMC Women’s Health.

[41] Informationen für Angehörige—Endometriose-Vereinigung Deutschland e.V. https://www.endometriose-vereinigung.de/informationen-fuer-angehoerige/.

[42] Zale M., Lambert E., LaNoue M.D., Leader A.E. (2020). Shedding Light on Endometriosis: Patient and Provider Perspectives on a Challenging Disease. J. Endometr. Pelvic Pain Disord..

[43] van der Zanden M., Teunissen D.A.M., van der Woord I.W., Braat D.D.M., Nelen W.L.D.M., Nap A.W. (2020). Barriers and Facilitators to the Timely Diagnosis of Endometriosis in Primary Care in The Netherlands. Fam. Pract..

[44] Frayne J., Milroy T., Simonis M., Lam A. (2023). Challenges in Diagnosing and Managing Endometriosis in General Practice. Aust. J. Gen. Pr..

[45] Grundström H., Hammar Spagnoli G., Lövqvist L., Olovsson M. (2020). Healthcare Consumption and Cost Estimates Concerning Swedish Women with Endometriosis. Gynecol. Obstet. Investig..

[46] Márki G., Vásárhelyi D., Rigó A., Kaló Z., Ács N., Bokor A. (2022). Challenges of and Possible Solutions for Living with Endometriosis: A Qualitative Study. BMC Women’s Health.

[47] Wren G., Mercer J. (2022). Dismissal, Distrust, and Dismay: A Phenomenological Exploration of Young Women’s Diagnostic Experiences with Endometriosis and Subsequent Support. J. Health Psychol..

[48] Roullier C., Sanguin S., Parent C., Lombart M., Sergent F., Foulon A. (2021). General Practitioners and Endometriosis: Level of Knowledge and the Impact of Training. J. Gynecol. Obstet. Hum. Reprod..

[49] Quibel A., Puscasiu L., Marpeau L., Roman H. (2013). Les Médecins Traitants Devant Le Défi Du Dépistage et de La Prise En Charge de l’endométriose: Résultats d’une Enquête. Gynécologie Obs. Fertil..

[50] Brauer L., de Cruppé W., Geraedts M. (2025). “Take Me Seriously”: A Qualitative Interview Study Exploring Healthcare Experiences of Endometriosis Patients. PLoS ONE.

[51] Chapron C., Marcellin L., Borghese B., Santulli P. (2019). Rethinking Mechanisms, Diagnosis and Management of Endometriosis. Nat. Rev. Endocrinol..

[52] Evans S., Villegas V., Dowding C., Druitt M., O’Hara R., Mikocka-Walus A. (2022). Treatment Use and Satisfaction in Australian Women with Endometriosis: A Mixed-Methods Study. Intern. Med. J..

[53] Timkova V., Mikula P., Katreniakova Z., Howick J., Nagyova I. (2025). Assessing Healthcare Needs in Endometriosis: A Scoping Review. Psychol. Health.

[54] Wilkinson R., Wynn-Williams M., Jung A., Berryman J., Wilson E. (2021). Impact of a Persistent Pelvic Pain Clinic: Emergency Attendances Following Multidisciplinary Management of Persistent Pelvic Pain. Aust. N. Z. J. Obstet. Gynaecol..

[55] Young K., Fisher J., Kirkman M. (2019). “Do Mad People Get Endo or Does Endo Make You Mad?”: Clinicians’ Discursive Constructions of Medicine and Women with Endometriosis. Fem. Psychol..

[56] Lukas I., Kohl-Schwartz A., Geraedts K., Rauchfuss M., Wölfler M.M., Häberlin F., Von Orelli S., Eberhard M., Imthurn B., Imesch P. (2018). Satisfaction with Medical Support in Women with Endometriosis. PLoS ONE.

[57] Arena A., Degli Esposti E., Orsini B., Moro E., Del Forno S., Cocchi L., Altieri M., Casadio P., Seracchioli R. (2023). Endometriosis in the Time of Internet: How Web Navigation Affects Women with Endometriosis. Ann. Med..

[58] Young K., Fisher J., Kirkman M. (2020). Partners Instead of Patients: Women Negotiating Power and Knowledge Within Medical Encounters for Endometriosis. Fem. Psychol..

[59] Nicola M., Correia H., Ditchburn G., Drummond P.D. (2022). Defining Pain-Validation: The Importance of Validation in Reducing the Stresses of Chronic Pain. Front. Pain Res..

[60] Paredes K.M., Afuape N.O., Vargas P.A. (2025). I Wish I Had Known: A Preliminary Study of the Journey of People with Early Onset Endometriosis in U.S.. J. Endometr. Uterine Disord..

[61] Eriksen A.A., Fegran L., Fredwall T.E., Larsen I.B. (2023). Patients’ Negative Experiences with Health Care Settings Brought to Light by Formal Complaints: A Qualitative Metasynthesis. J. Clin. Nurs..

[62] Hållstam A., Stålnacke B.M., Svensén C., Löfgren M. (2018). Living with Painful Endometriosis—A Struggle for Coherence. A Qualitative Study. Sex. Reprod. Healthc..

[63] Young K., Fisher J., Kirkman M. (2016). Endometriosis and Fertility: Women’s Accounts of Healthcare. Hum. Reprod..

[64] Facchin F., Saita E., Barbara G., Dridi D., Vercellini P. (2018). “Free Butterflies Will Come out of These Deep Wounds”: A Grounded Theory of How Endometriosis Affects Women’s Psychological Health. J. Health Psychol..

